# Mitochondrial Dysfunction in Individuals with Diabetic Kidney Disease: A Systematic Review

**DOI:** 10.3390/cells11162481

**Published:** 2022-08-10

**Authors:** Nicole Flemming, Laura Pernoud, Josephine Forbes, Linda Gallo

**Affiliations:** 1 School of Medicine and Dentistry, Griffith University, Birtinya 4556, Australia; 2 Faculty of Medicine, University of Queensland, Brisbane 4072, Australia; 3 Mater Research Institute, The University of Queensland (MRI-UQ), Brisbane 4072, Australia; 4 School of Health and Behavioural Sciences, University of the Sunshine Coast, Maroochydore 4558, Australia; 5 School of Biomedical Sciences, University of Queensland, Brisbane 4072, Australia

**Keywords:** mitochondrial dysfunction, mitochondrial dynamics, mitophagy, mitochondrial fission, mitochondrial fusion, oxidative stress, diabetic kidney disease, systematic review

## Abstract

Mitochondrial dysfunction is implicated in the pathogenesis of diabetic kidney disease (DKD). Compared to the vast body of evidence from preclinical in vitro and in vivo studies, evidence from human studies is limited. In a comprehensive search of the published literature, findings from studies that reported evidence of mitochondrial dysfunction in individuals with DKD were examined. Three electronic databases (PubMed, Embase, and Scopus) were searched in March 2022. A total of 1339 articles were identified, and 22 articles met the inclusion criteria. Compared to non-diabetic controls (NDC) and/or individuals with diabetes but without kidney disease (DC), individuals with DKD (age ~55 years; diabetes duration ~15 years) had evidence of mitochondrial dysfunction. Individuals with DKD had evidence of disrupted mitochondrial dynamics (11 of 11 articles)*,* uncoupling (2 of 2 articles), oxidative damage (8 of 8 articles), decreased mitochondrial respiratory capacity (1 of 1 article), decreased mtDNA content (5 of 6 articles), and decreased antioxidant capacity (3 of 4 articles) compared to ND and/or DC. Neither diabetes nor glycemic control explained these findings, but rather presence and severity of DKD may better reflect degree of mitochondrial dysfunction in this population. Future clinical studies should include individuals closer to diagnosis of diabetes to ascertain whether mitochondrial dysfunction is implicated in the development of, or is a consequence of, DKD.

## 1. Introduction

**Figure 1:**
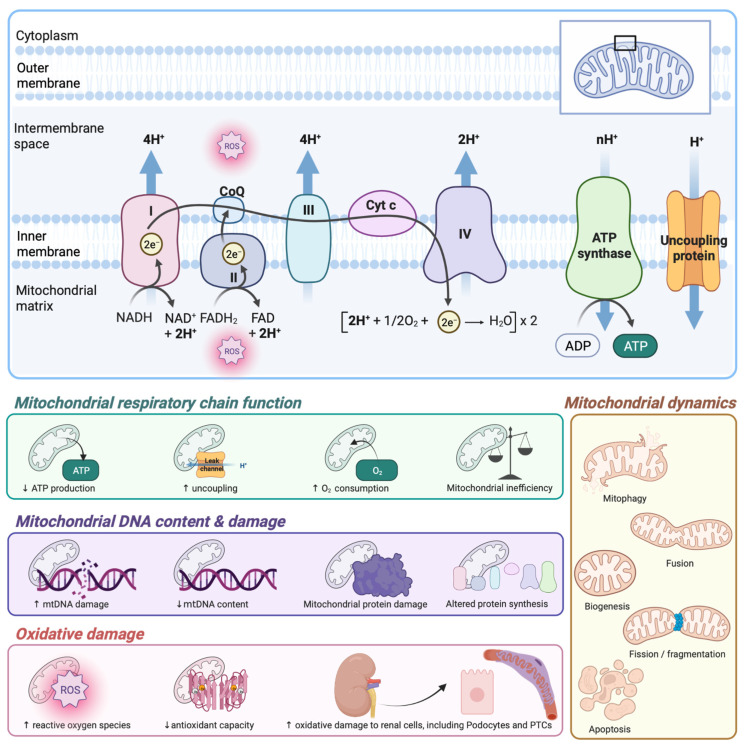
Potential pathways of mitochondrial dysfunction contributing to and/or exacerbating disturbances in renal oxygenation and energetics in diabetes. ADP, adenosine diphosphate; ATP, adenosine triphosphate; CoQ, coenzyme Q; Cyt C, cytochrome C; e^−^, electrons; FAD, flavin adenine dinucleotide (oxidised); FADH_2_, flavin adenine dinucleotide (hydroquinone); H^+^, hydrogen ions; H_2_O, water; mtDNA, mitochondrial deoxyribonucleic acid; NAD^+^, nicotinamide adenine dinucleotide (oxidised); NADH, nicotinamide adenine dinucleotide (reduced); O_2_, oxygen; PTCs, proximal tubule cells; ROS, reactive oxygen species. Image created with BioRender.com.

Diabetic kidney disease (DKD) is a microvascular complication that affects 27–40% of individuals with diabetes [1]. While DKD is driving an increase in the global prevalence of end-stage renal disease (ESRD), it is also a major contributor to premature death, resultant from cardiovascular disease [2]. Mitochondrial dysfunction, a deleterious change to mitochondrial structure and function, has been implicated in the development and progression of DKD [3,4,5]. Several mitochondrial pathways are altered in diabetes, including mtDNA content, mitochondrial dynamics and uncoupling, mitochondrial respiration, oxidative damage, and antioxidant capacity, and likely contribute to and/or exacerbate disturbances in renal oxygenation and energetics (Figure 1).

Mitochondria are intracellular organelles that produce the majority of cellular adenosine triphosphate (ATP) via the tricarboxylic acid (TCA) cycle and oxidative phosphorylation and are abundant in cells and tissues with high energetic requirements. During aerobic ATP production, electrons that escape from the mitochondrial respiratory chain can form reactive oxygen species (ROS), particularly when the membrane potential is high due to increased flux through the respiratory chain. Given the high energy requirements of the kidney, particularly the proximal tubules, the kidney contains the second highest density of mitochondria [6]. The kidney receives approximately 20% of the total cardiac output to sustain renal ATP supply [3,6,7]. Accordingly, the kidney is susceptible to mitochondrial dysfunction and decreased oxidative capacity, where disturbances in oxygenation and energetics are frequently described in the diabetic kidney [3].

Mitochondria contain their own circular genome (mitochondrial DNA; mtDNA) which encodes essential mitochondrial proteins [8]. Due to the structure of, and proximity to, the respiratory chain, mtDNA is susceptible to damage mediated by oxidation, glycation, and inflammation. Damage to mtDNA results in mutations and deletions [9], production of defective mitochondrial proteins which perpetuate ROS production [10], compromised ATP production [11], and altered tissue oxygenation [3,4]. Additionally, extracellular mtDNA can be released in response to signals of cellular stress or damage as free circulating mtDNA or packaged into extracellular vesicles (e.g., apoptotic bodies, microvesicles, or exosomes) [12]. Changes to mtDNA content, commonly expressed as mitochondrial to nuclear genome ratio, have been reported as a biomarker of mitochondrial dysfunction in a range of human pathologies [11]. However, the relationship between mtDNA content and DKD remains to be adequately defined. 

Mitochondria dynamics regulate mitochondrial function and capacity to produce ATP and are altered by diabetes [4,5,13]. Fission is essential for maintaining mitochondrial health by quality control, yielding defective daughter mitochondria for targeted mitophagy. Dysfunctional mitochondria not degraded by mitophagy produce ROS at increased rates and are disposed to release of Cytochrome C and apoptosis-inducing factor [14], which are part of essential processes that trigger cell death. Mitochondrial fission is largely mediated by cytosolic dynamin-related protein-1 (DRP1), in addition to mitochondrial fission protein-1 (FIS1), A-kinase anchoring protein 1 (AKAP1), and other mitochondrial dynamics proteins [9,15,16]. Mitochondrial fragmentation is a hallmark of apoptosis and occurs as a result of increased fission and decreased fusion [15]. A complex synergistic relationship exists between fission and apoptosis, where extreme stresses that cause apoptosis also induce excessive fission [9]. As an example, accumulation of mitochondrial ROS results in the translocation of BAX (proapoptotic protein) and subsequently the release of Cytochrome C into the cell cytosol, triggering apoptosis [17]. Conversely, mitochondrial fusion enables mitochondria to maximise oxidative capacity, especially during pathological stress [9]. Fusion of the outer mitochondrial membranes is mediated by mitofusin-1 (MFN1) and -2 (MFN2), while fusion of the inner membranes and maintenance of the mitochondrial crista structure is mediated by optic atrophy-1 (OPA1) protein [18,19]. 

Mitophagy, the breakdown and turnover of dysfunction mitochondria, is closely linked with fission and fusion pathways [9]. Mitochondrial depolarisation precedes protein translocation that flags mitochondria for mitophagy via the expression of PTEN induced kindase-1 (PINK1) and Parkin [14]. In healthy mitochondria, PINK1 is imported into the inner mitochondrial membrane where it is degraded by Presenilins-associated rhomboid-like protein (PARL). However, in damaged mitochondria, membrane potential loss prevents PINK1 translocation and degradation, resulting in its accumulation on the outer mitochondrial membrane and indicates mitochondrial damage. The process results in the recruitment of cytosolic Parkin which ubiquitinates the outer membrane, thereby targeting the organelle for mitophagy [9].

Mitochondrial biogenesis occurs during cell division and increases mitochondrial mass to regulate metabolism, ATP production, and response to oxidative stress [20]. The master regulators of biogenesis are two transcriptional coactivators, peroxisome proliferator-activated receptor (PPAR)-g coactivator (PGC)-1a and PGC-1b, which activate mitochondrial transcription factor A (TFAM) via nuclear respiratory factor (NRF)-1, as well as several oxidative phosphorylation genes [21,22]. While PGC-1a-mediated increases in biogenesis can raise capacity for respiration and ATP production, this often concomitantly increases ROS. However, in healthy mitochondria, PGC-1a also regulates transcription of antioxidants and detoxifying enzymes including superoxide dismutase-2 (SOD2), uncoupling protein-2 (UCP2), and glutathione peroxidase-1 (GPX1). Interestingly, decreased renal PGC-1a expression is commonly observed in humans and in murine models with acute and chronic kidney disease, while increased expression can restore energy deficits and is considered a protective mechanism against chronic kidney disease (CKD) [21]. 

Hyperglycaemia is postulated to increase production of ROS, contributing to oxidative damage and the pathogenesis of diabetic vascular complications. Superoxide can react with nitric oxide to form peroxynitrite, modifying mitochondrial proteins and contributing to vasoconstriction, decreased renal blood flow and oxygen delivery [23]. Superoxide can also be converted to hydrogen peroxide by superoxide dismutase 1 (SOD1) or SOD2. Hydrogen peroxide can freely diffuse into the cytosol and mediate extra-mitochondrial damage or be converted to water via GPX1 or other highly reactive molecules. Oxidative stress can increase the conversion of deoxyguanosine to 8-hydroxyguanosine (8-oxo-dG; marker of oxidative stress) in DNA and may result increased mtDNA damage [24,25]. Furthermore, altered mitochondrial dynamics, including excessive fission and/or decreased fusion can perpetuate ROS production [17]. Indeed, a plethora of studies in individuals with diabetes have investigated markers of oxidative stress [26,27,28,29,30,31,32,33,34] and compromised antioxidant capacity [28,35,36]. While oxidative stress is recognised as a common pathway to diabetes and vascular complications [37,38], studies demonstrating end-organ protection with antioxidant therapies are limited [3,5], suggesting that pathways other than, or in addition to, oxidative stress may prevail as pathological mediators in DKD. 

Mitochondrial uncoupling occurs when tissue-dependent uncoupling proteins (UCPs) bind complex V and facilitate electron influx into the mitochondrial matrix; however, chronic overexpression of UCPs can increase superoxide production [39]. Furthermore, systemic exploitation of uncoupling can increase mitochondrial oxygen consumption resulting in localised hypoxia and as such may contribute to or exacerbate renal damage. Indeed, increased oxygen consumption is evidenced in the diabetic kidney [40,41,42] and renal cells cultured in diabetic-like environments [20,43,44], which may be attributed, at least in part, to dysregulated mitochondrial respiration as a result of altered glucose, lipid and amino acid profiles [5]. Increased mitochondrial oxygen consumption due to altered metabolic environments is postulated to precede glomerular damage and contribute to renal hypoxia and fibrosis in the diabetic kidney, though the mechanisms remain unknown [4,40]. 

While it is generally accepted that mitochondrial dysfunction plays a role in the development and progression of DKD, most evidence has come from cell-culture and pre-clinical studies, and direct evidence from clinical studies is limited. Accordingly, the aim of this study was to systematically review the evidence for systemic and kidney-specific mitochondrial dysfunction in human DKD, and to determine whether this area requires further research.

## 2. Methods

**Figure 2:**
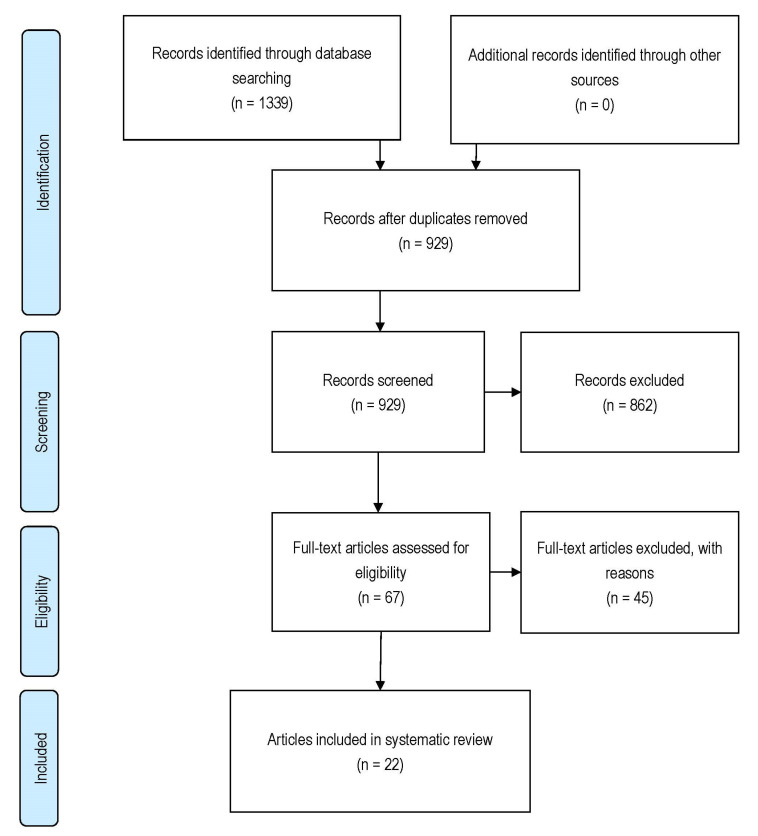
Study identification, screening, eligibility, and selection process.

A prospective protocol for this systematic review was developed a priori, where the search terms and inclusion/exclusion criteria were chosen to capture all relevant articles. A computer-aided database searching occurred on 30 March 2022, utilising the following three databases: PubMed, Embase, and Scopus. The search strategy was based on free text terms, using the text words “diabetic kidney disease*” or “diabetic nephropathy*” or “diabetic nephropathies” and “mitochondria*” or “mitochondrio*”. Databases were searched with external limiters; however, the exact external limiters differed among databases due to the availability of search options. The references cited in relevant publications were also cross-referenced to identify additional studies that may have been missed in the initial database search. Figure 2 depicts the articles identified during the search process.

The search strategy was designed to capture all articles that report the association between mitochondrial dysfunction and DKD. Articles were initially included if they (i) included sample/s (biopsy, cells, blood, plasma, serum, urine) from individuals with DKD secondary to type 1 or type 2 diabetes; (ii) included sample/s from a control comparator (no diabetes without kidney disease and/or diabetes without kidney disease); and (iii) reported at least one outcome related to mtDNA content, mtDNA damage, mitochondrial dynamics (fission, fragmentation, fusion, mitophagy, autophagy, biogenesis), mitochondrial uncoupling, mitochondrial membrane potential, respiratory capacity, oxidative stress/damage, or antioxidant capacity. Exclusion criteria were (i) articles without adequate findings to evidence mitochondrial dysfunction; (ii) cell culture studies that did not include sample/s from individuals with diagnosed kidney disease secondary to type 1 or type 2 diabetes; (iii) animal studies; (iv) intervention studies; (v) review articles; (vi) unpublished studies, abstracts, case reports or dissertations; (vii) non-peer reviewed articles, book chapters, conference abstracts, or protocols; (viii) non-English language articles. 

Following the database search, duplicate records were removed, and title and abstract screening was performed by two authors (N.B.F. and L.A.G.) for eligibility. Following this, full text articles were identified and independently assessed by two reviewers (N.B.F. and L.E.P.) for eligibility and inclusion. Data extraction relating to study design, population, outcome measures, and methodologies were completed by two authors (N.B.F. and L.E.P.) using a standardised data extraction spreadsheet. An updated search was performed prior to submission, and after duplicates were removed an additional 264 records underwent title and abstract screening (N.B.F.), 3 articles were eligible for full-text screening (N.B.F.), and 2 articles were included. Extracted data included author/s, year of publication, sample size, population characteristics (e.g., HbA1_c_, albuminuria, eGFR, fasting blood glucose, BMI, serum creatinine), methods, and outcomes directly or indirectly related to mitochondrial function. 

Two authors (N.B.F. and L.E.P.) independently assessed the risk bias using the BIOCROSS scale, modified for the current review (Table A1). Both reviewers rated the eligibility of articles and provided a score between zero and two for each of the ten “Issues to Consider” (IC). Mentioning all ICs was awarded a score of two, if one or two ICs were missing a score of one was awarded, and zero was awarded if no ICs were mentioned. Any disagreements between the two reviewers scores were identified and discussed before an agreement was reached. 

We reviewed all studies that included individuals with DKD and presented biochemical markers that could indicate mitochondrial dysfunction. The primary objective of this systematic review was to report and compare markers of mitochondrial dysfunction between individuals with DKD and those without DKD. 

## 3. Results

Figure 2 illustrates the study identification, screening, eligibility, and selection process. A total of 1339 articles were identified through three database searches. After removing duplicate publications, 929 titles and abstracts were screened for eligibility, and 862 articles were excluded based on the inclusion/exclusion criteria. After full-text review of 67 articles, an additional 45 articles were excluded for not meeting the inclusion criteria. A total of 22 articles were eligible for inclusion. BIOCROSS analysis indicated a moderate quality of studies, with an average score of 62.0 ± 9.3% (Table 1). 

The population characteristics varied across each study cohort, and a summary is provided in Table 1. Of the 22 articles included in the systematic review, two articles included only individuals with T1DM [45,46], 10 articles included only individuals with T2DM [17,47,48,49,50,51,52,53,54,55], three articles included both T1DM and T2DM [56,57,58], and seven articles did not specify [59,60,61,62,63,64,65]. Most studies included middle-to-older age subjects with a long duration (~15 years) of diabetes. A range of mitochondrial outcomes were reported, and findings were classified into one of the following four categories: mtDNA content and damage, mitochondrial dynamics, oxidative damage and antioxidants, and mitochondrial respiration, uncoupling and membrane potential.

**Table 1:** Summary of population characteristics.

Study	QR (%)	Sample	Group	DM Type	N	Age (Years)	BMI (kg/m^2^)	DM Duration (Years)	HbA_1c_ (%)	Proteinuria (See Footnotes)	eGFR (mL/min/1.73 m^2^)	SCr (µmol/L)
Al-Kafaji [47]	70	Peripheral blood	NDC	n/a	50	56.0 ± 5.2	24.2 ± 4.1	n/a	4.9 ± 0.7	0.9 ± 0.4 ^a^	104.3 ± 13.2	54.7 ± 11.7
			DC	T2	50	62.0 ± 10.7 *	24.8 ± 4.4	15.0 ± 4.5	8.9 ± 2.2 *	0.9 ± 0.5 ^a^	94.0 ± 8.1	64.7 ± 15.7 *
			DKD	T2	50	64.5 ± 6.3 *	26.3 ± 5.2 *	17.5 ± 4.6	9.3 ± 1.8 *	28.3 ± 12.0 ^a^ * ^†^	66.0 ± 14.8 * ^†^	119.2 ± 45.8 * ^†^
Cao [48]	70	Plasma & urine	NDC	n/a	35	53.0 ± 10.8	23.5 ± 1.8	n/a	4.8 ± 0.6	16.7±13.6 ^b^	—	72.4 ± 14.2
			DC + DKD	T2	42	58.0 ± 12.0 ^c^	24.7 ± 1.6 ^c^ *	9.5 ± 8.2 ^c^	9.6 ± 2.4 ^c^ *	150.0 ± 155.8 ^b c^ *	—	99.3 ± 63.5 ^b^ *
Czajka [56]	80	Peripheral blood & PBMCs	NDC	n/a	39	52.0 ± 25.0	23.0 ± 4.0	n/a	—	—	—	—
			DC	T1 + T2	45	49.0 ± 14.0	27.0 ± 4.0 *	22.0 ± 10.0	10.0 ± 9.0	0.8 ± 0.6 ^a^	103.0 ± 21.0	—
			DKD	T1 + T2	83	61.0 ± 14.0 ^††^	30.0 ± 7.0 ** ^††^	22.0 ± 13.0	8.0 ± 1.0	18.0 ± 50.0 ^a ††^	66.0 ± 34.0 ^††^	—
Dieter [45]	70	Plasma & urine	DC	T1	17	24.2 ± 5.5	23.2 ± 3.3	15.6 ± 5.0	8.6 ± 0.9	6.1 [3.3–9.3] ^d u^	123.0 [112.5–126.0] ^u^	0.7 [0.6–0.9] ^e u^
			DKD^moderate^	T1	12	21.8 ± 4.1	22.6 ± 1.9	15.3 ± 5.8	10.5 ± 2.1 ^††^	76.9 [36.0–168.7] ^d u^	112.0 [87.7–127.7] ^u^	0.9 [0.8–1.2] ^e u^
			DKD^severe^	T1	11	30.6 ± 5.7 ^††^ ˆˆ	23.7 ± 3.8	23.7 ± 5.2 ^††††^ ˆˆˆˆ	10.2 ± 1.5 ^††^	740.7 [410.3–2551.8] ^d u^	16.0 [6.0–87.5] ^u^	4.5 [1.0–8.1] ^e u^
Ding [64]	50	Biopsy	NDC	n/a	6	53.2 ± 8.40	—	—	—	—	—	—
			DKD	n/s	6	57.0 ± 9.27	—	—	—	—	—	—
Ding [65]	50	Biopsy	NDC	n/a	6	51.0 ± 9.59	—	—	—	—	—	—
			DKD	n/s	6	53.5 ± 8.71	—	—	—	—	—	—
Han [59]	55	Biopsy	NDC	n/a	15	—	23.0 (3.4)	n/a	4.8 (0.5)	3.2 (1.3) ^f^	—	92.3 (8.9)
			DKD	n/s	15	—	24.9 (4.0)	—	8.1 (0.6) *	4.4 (1.1) ^f^ *	—	94.2 (7.9)
Horne [60]	70	Biopsy	NDC	n/a	12	64.0 [54.0–75.0]	24.0 [21.0–38.0]	n/a	—	—	87.0 [74.0–102.0]	—
			DKD^early^	n/s	7	59.0 [42.0–69.0]	32.0 [25.0–33.0]	—	7.1 [6.6–8.3]	—	72.0 [70.0–77.0]	—
			DKD^late^	n/s	17	62.0 [46.0–68.0]	31.0 [29.0–35.0]	—	6.6 [6.3–9.1]	—	26.0 [19.0–36.0] **	—
Jiang [17]	70	Serum & PBMCs	NDC	n/a	65	49.7 (1.5)	—	n/a	—	—	—	—
			DC	T2	48	54.0 (2.3)	23.4 (0.5)	5.0 {9.4}	—	1.6 {1.4} ^h^	100.8 (2.4)	—
			DKD	T2	60	53.4 (1.5)	24.2 (0.4)	8.0 {7.8} ^†^	—	278.2 {430.5} ^h †††^	60.8 (4.0) ^†††^	—
		Biopsy	NDC	n/a	15	—	—	—	—	—	—	—
			DKD	n/s	14	—	—	—	—	—	—	—
Li [49]	60	Urine	NDC	n/a	20	58.0 [42.5–66.5]	—	n/a	5.2 [5.0–5.8]	4.3 [3.3–6.4] ^i^	91.3 [85.4–104.5]	—
			DC	T2	25	61.0 [47.3–68.0]	—	—	6.9 [6.8–7.2] *	7.7 [4.5–13.1] ^i^ *	84.2 [72.4–98.7]	—
			DKD	T2	24	59.5 [43.0–67.3]	—	—	7.1 [6.9–7.2] *	978.3 [90.7–2588.3] ^i^ * ^†^	57.8 [30.8–77.2] * ^†^	—
		Biopsy	NDC	n/a	5	—	—	n/a	—	—	—	—
			DC	T2	5	—	—	—	—	—	—	—
			DKD	T2	5	—	—	—	—	—	—	—
Li [61]	50	Biopsy	NDC	n/a	14	—	—	n/a	—	—	—	—
			DKD	n/s	7	—	—	—	—	—	—	—
Li [50]	75	Biopsy	NDC	n/a	10	—	—	n/a	—	0.1 ± 0.05 ^f^	115.3 ± 10.0	57.9 ± 11.8 ^j^
			DKD	T2	33	—	—	—	—	3.2 ± 0.8 ^f^ ***	62.7 ± 11.8 ***	115.6 ± 27.5 ^j^ ***
Li [51]	75	Biopsy	NDC	n/a	10	—	26.5 ± 1.4	n/a	—	0.2 ± 0.1 ^f^	116.3 ± 9.4	59.0 ± 10.6 ^j^
			DKD	T2	34	—	32.1 ± 2.2 ***	—	—	3.8 ± 2.5 ^f^ ***	64.5 ± 36.4 ***	137.7 ± 122.3 ^j^ *
Ma [62]	80	Biopsy	NDC	n/a	6	57.8 ± 3.7	—	n/a	—	0.1 ± 0.02 ^g^	—	70.0 ± 9.0
			DKD	n/s	31	49.9 ± 2.5	—	—	—	5.4 ± 0.7 ^g^ ***	—	108.0 ± 8.0 *
Malik [52]	75	Peripheral blood	NDC	n/a	21	50.0 ± 12.0	26.0 ± 5.0	n/a	—	6.3 [1.6–28.0] ^k^	—	0.9 ± 0.1 ^l^
			DC	T2	20	53.0 ± 11.0	25.0 ± 3.0	—	—	5.3 [1.6–23.0] ^m^	—	1.0 ± 0.2 ^l^
			DKD	T2	21	54.0 ± 9.0	25.0 ± 3.0	—	—	3.4 [1.6–10.7] ^n^	—	1.5 ± 0.5 ^l^
Mohammedi [46]	80	Plasma	DC	T1	131	—	—	—	—	—	—	—
			DKD^incipient^	T1	83	—	—	—	—	—	—	—
			DKD^advanced^	T1	167	—	—	—	—	—	—	—
Qi [57]	60	Biopsy	NDC	n/a	6	37.0 ± 18.5	24.1 ± 2.5	n/a	—	—	—	—
			DKD^mild-moderate^	T2	7	63.2 ± 15.7	29.1 ± 6.6	—	—	—	—	—
			DKD^mild-moderate^	T1	1	28.0	28.4	—	—	—	—	—
			DKD^moderate-severe^	T2	5	46.8 ± 10.1	—	—	—	—	—	—
			DKD^advanced^	T2	1	51.0	—	—	—	—	—	—
		Urine	DC	n/s	22	54.8 (3.9)	—	—	—	0.1 (0.0) ^o^	116.4 (14.8)	—
			DKD^non-progressive^	n/s	14	65.9 (3.7)	—	—	—	0.3 (0.2) ^o^	51.7 (4.9) ^††† t^	—
			DKD^intermediate^	n/s	23	68.3 (2.2)	—	—	—	1.3 (0.5) ^o^	46.3 (3.7) ^††† t^	—
			DKD^progressive^	n/s	12	63.8 (4.5)	—	—	—	2.8 (1.1) ^o^	39.3 (3.6) ^††† t^	—
Qin [63]	80	Plasma & biopsy	NDC	n/a	15	45.9 ± 5.5	22.6 ± 3.4	n/a	5.0 ± 0.9	—	98.6 ± 8.2	72.3 ± 15.0
			DKD	n/s	20	55.7 ± 8.3 *	23.8 ± 3.0	—	6.7 ± 0.9 *	76.6 ± 40.5 ^p^	90.3 ± 14.5	74.0 ± 19.7
Sas [53] **^i^**	65	Urine	NDC	n/a	10	50 [48.5–51.3]	—	n/a	—	18.5 [6.9–37.3] ^k^	91.5 [73.8–103.3]	0.8 [0.8–1.0] ^q^
			DC	T2	10	52 [42.8–65.0]	—	22.0 [13.3–26.3]	7.1 [6.9–8.6]	6.5 [0.0–11.3] ^k^	91.8 [76.9–102.8]	0.8 [0.7–0.9] ^q^
			DKD	T2	10	67 [52.8–74.5]	—	17.5 [11.5–23.5]	7.7 [6.6–8.2]	33.0 [4.5–56.0] ^k^	93.3 [71.7–103.9]	0.8 [0.7–1.0] ^q^
Sharma [58]	85	Urine & plasma	NDC	n/a	23	37.7	—	n/a	—	—	—	—
			DC	T1	32	45.7 ± 10.4	25.5 ± 3.5	31.5 [25.0–37.2]	7.9 ± 1.0	0.08 [0.03–0.8]	106.0 ± 25.8	0.8 ± 0.2 ^l^
			DC	T2	41	59.1 ± 6.8	24.8 ± 3.7	11.0 [8.0–15.0]	8.4 ± 1.3	0.09 [0.05–0.3]	79.8 ± 15.2 ^‡‡‡ t^	0.9 ± 0.2 ^l^
			DKD^screening^	T2	24	64.2 ± 7.8	34.2 ± 6.2	16.0 [10.0–21.0]	7.2 ± 1.1	0.8 [0.12–1.3]	35.5 ± 10.9 ^‡‡‡ §§§ t^	2.2 ± 0.6 ^l^
			DKD^validation^	T1	61	60.6 ± 11.9	32.4 ± 7.2	30.0 [24.2–37.8] ^r^	7.4 ± 1.3	0.21 [0.1–1.1]	36.0 ± 13.4 ^‡‡‡ §§§ t^	2.2 ± 0.9 ^l^
				T2				15.0 [9.5–23.0] ^s^				
		Biopsy-1	NDC	n/a	5	—	—	n/a	—	—	—	—
			DKD	n/s	5	—	—	—	—	—	—	—
		Biopsy-2	NDC	n/a	8	—	—	n/a	—	—	—	—
			DKD	n/s	14	—	—	—	—	—	—	—
Suzuki [54]	55	Muscle biopsy	NDC	n/a	7	—	—	n/a	—	—	—	—
			DC	T2	6	—	—	—	—	—	—	—
			DKD^micro^	T2	4	—	—	—	—	—	—	—
			DKD^macro^	T2	7	—	—	—	—	—	—	—
			DKD^crf^	T2	5	—	—	—	—	—	—	—
Zhou [55]	65	Biopsy	NDC	n/a	8	—	—	n/a	—	—	—	—
			DC	T2	7	—	—	—	—	—	—	—
			DKD	n/s	8	—	—	—	—	—	—	—

Data are presented as mean ± SD, mean (SEM), median {interquartile range}, median [25–75th percentiles]. *p*-values are relative to NDC and DC within each study; * *p* ≤ 0.05 vs. NDC, ** *p* ≤ 0.01 vs. NDC, *** *p* ≤ 0.001 vs. NDC, ^†^ *p* ≤ 0.05 vs. DC, ^††^ *p* ≤ 0.01 vs. DC, ^†††^ *p* ≤ 0.001 vs. DC, ^††††^ *p* ≤ 0.0001 vs. DC, ^‡‡‡^ *p* ≤ 0.001 vs. DC (T1), ^§§§^ *p* ≤ 0.001 vs. DC (T2), ˆˆ *p* ≤ 0.01 vs. DKD (moderate), ˆˆˆˆ *p* ≤ 0.0001 vs. DKD (moderate). ^(a)^ uACR (mg/mmol); ^(b)^ urine albumin (mg/L); ^(c)^ DC and DKD groups combined for participant characteristics; ^(d)^ UAE (mg/g); ^(e)^ μg/dL; ^(f)^ urine protein (g/24 h); ^(g)^ urine protein (g/L); ^(h)^ uACR (g/mol); ^(i)^ uACR (mg g^−1^); ^(j)^ uM; ^(k)^ uACR (mg/g); ^(l)^ mg/dL; ^(m)^ 24 h UAE (mg/d); ^(n)^ 24 h urine protein excretion (g/d); ^(o)^ urine protein:creatinine (mg/mg); ^(p)^ AER (mg/24 h); ^(q)^ Scr (mg/dL); ^(r)^ T1DM diabetes duration; ^(s)^ T2DM diabetes duration; ^(t)^ statistics not reported in manuscript, calculated using extracted data; ^(u)^ statistics not reported. AER, albumin excretion rate; BMI, body mass index; crf, chronic renal failure; DC, diabetes control; DKD, diabetic kidney disease; DM, diabetes mellitus; eGFR, estimated glomerular filtration rate; h, hour; HbA_1c_, glycated haemoglobin; macro, macroalbuminuria; micro, microalbuminuria; n, number; n/a, not applicable; —, not reported; NDC, non-diabetic control; ns, not specified; PBMCs, peripheral blood mononuclear cells; QR, quality rating; sCr, serum creatinine; T1, type 1; T2, type 2; uACR, urinary albumin creatinine ratio.

### 3.1. mtDNA Content and Damage

Six studies investigated mtDNA content in individuals with DKD, of which four studies were in blood [17,47,52,56], one in urine [58], and one study in both [48]. The mtDNA values reported varied between studies, likely due to different methodology, though the majority (5 of 6 studies) reported decreased mtDNA content in individuals with DKD. These findings are summarised in Table 2. 

In DC versus NDC individuals, circulating mtDNA content was altered, though the findings were inconsistent. Studies reported that circulating mtDNA was increased [56], decreased [47], or unchanged [17,48,52]. In people with DKD, one study reported that circulating mtDNA was elevated compared to NDC and DC [52], but in all others, circulating mtDNA content was decreased compared to NDC [47,48] and/or DC [17,47,56]. Interestingly, one study reported that individuals with diabetes (i.e., DC and DKD groups combined, DM (*n* = 42)) had decreased circulating mtDNA compared to NDC [48]. However, when the cohort was further stratified by the presence or absence of kidney disease in diabetes (i.e., NDC (*n* = 35), DC (uACR < 0.05 g/24 h; *n* = 23), DKD (>0.05 g/24 h; *n* = 19)) mtDNA content was significantly decreased only in individuals with DKD but remained unchanged in the DC group, compared to NDC [48]. When the participant cohort was further stratified by clinical uACR conventions (i.e., DC with normoalbuminuria (*n* = 50), DKD with microalbuminuria (*n* = 29), and DKD with macroalbuminuria (*n* = 21)), mtDNA was lower in individuals with macroalbuminuria (16.7 ± 2.9) compared to microalbuminuria (23.8 ± 3.5, *p* < 0.01) and normoalbuminuria (35.11 ± 5.4, *p* < 0.01) [47]. In support of this, another study reported an inverse correlation between plasma mtDNA and urinary albumin excretion (*r* = −0.251, *p* < 0.05) [48].

Two studies reported urinary mtDNA content, though the methodology was vastly different [48,58]. One study reported urinary mtDNA/creatinine ratio was increased in individuals with diabetes versus NDC, and positively correlated with urinary albumin excretion (*r* = 0.0242, *p* < 0.05) [48]. Another study reported mtDNA content in urinary exosomes was decreased in individuals with DKD compared to NDC [58].

Individuals with DKD had evidence of heteroplasmy and mtDNA damage. mRNA levels of mitochondrial encoded subunits, ND6, ND1, and COX3, were unchanged despite altered mtDNA content in individuals with diabetes in the presence and absence of DKD [56]. In one study, mtDNA relative amplification was decreased in individuals with diabetes (although not significant; ns) despite an increase in mtDNA content [56]. In another study, mtDNA was decreased compared to NDC (*p* < 0.0001) and DC (*p* = 0.005) [17].

### 3.2. Mitochondrial Dynamics

As mtDNA content and repair are largely influenced by mitochondrial dynamics, we profiled mitochondrial dynamics in individuals with DKD (Table 3). Mitochondrial fission/fragmentation was reported in four studies [17,56,59,62], fusion in three studies [17,56,64], biogenesis in six studies [49,50,55,56,58,61], apoptosis in three studies [17,58,60], and one study reported on mitophagy [56].

Individuals with DKD had evidence of increased mitochondrial fission/fragmentation and decreased fusion. Compared to NDC, DKD kidneys had increased mitochondrial fission proteins, DRP1 and FIS1 [17], percentage of dysmorphic mitochondria [17] and fragmentation in the proximal tubules [17,59], while the number of fragmented tubular cells were positively correlated with oxidative stress (r = 0.704, *p* < 0.01) [59]. Podocyte mitochondrial fragmentation, and colocalisation of glomerular AKAP1 and DRP1 expression, were increased compared to DC [62]. Conversely, nuclear-encoded mitochondrial mRNA, DRP1, in peripheral blood was decreased in diabetic individuals with and without DKD, although this was not significant [56]. Renal mitochondrial fusion protein, MFN2, was decreased in renal biopsies taken from individuals with DKD compared to NDC [17,64]. This is in contrast to another study reporting unchanged nuclear-encoded mitochondrial mRNA, MFN1 and MFN2, in peripheral blood, while OPA1 tended to decrease in DC and DKD individuals [56].

Individuals with DKD had greater percentage of apoptotic PBMCs and cells undergoing apoptosis in proximal tubules [17]. BAX and Cytochrome C levels were elevated in the renal tubule cytosol [17], as evidenced by immunostaining and semi-quantitative morphological analysis while renal mitochondrial Cytochrome C oxidase subunit II was decreased compared with NDC [58]. Glomerular KLF6 (Kruppel-like factor 6) was decreased in late-stage DKD, and podocyte-specific KLF6 was decreased in both early-stage and late-stage DKD, compared to NDC [60].

The gene expression of mitophagy markers PINK1 and PARK2 by qPCR was decreased in peripheral blood samples taken from individuals with diabetes, regardless of DKD status, compared to NDC (data not shown) [56]. However, individuals with DKD had increased PARK2 compared to DC [56].

Individuals with DKD had evidence of decreased capacity for renal mitochondrial biogenesis. Compared to NDC, PPARGC1A (encodes PGC1-α) mRNA transcription was downregulated in DKD glomeruli, and PGC1-α protein expression and expression of mitochondrial genes were decreased [61]. Similarly, renal PGC1-α and p-AMPK were decreased compared to NDC and DC [49], while renal PGC1-α mRNA expression was decreased (0.4-fold-change) compared with NDC [58]. Nuclear-encoded mitochondrial mRNA mitochondrial transcription factor A (TFAM) was increased compared to DC, while PGC1-α was unchanged [56]. Progranulin was decreased in renal cortical tissue compared to NDC and DC [55]. In contrast, one study demonstrated increased PGC1-α in renal biopsy samples taken from individuals with DKD [50]. 

### 3.3. Oxidative Damage and Antioxidants

Altered mitochondrial dynamics, mitochondrial uncoupling, and dysfunctional mitochondrial respiration contribute to oxidative damage. Eight studies included a measure of oxidative damage, and these findings are presented in Table 4. 

Individuals with DKD had increased markers of oxidative damage, including H_2_DCFDA [17], superoxide [59], Nox4 [17], nitrotyrosine [17], malondialdehyde [63], and AOPP [46,50,51]. In individuals with moderate-to-severe and advanced DKD, 8-oxoG accumulated in glomerular endothelial cells but not in synaptopodin-positive podocytes (data not shown) and was not detectable in DC glomeruli [57]. Urinary 8-oxoG/creatinine was increased in individuals with progressive DKD compared to DC and non-progressive DKD [57]. In muscle DNA, 8-OHdG content was higher in individuals with DKD and microalbuminuria, macroalbuminuria and chronic renal failure compared to NDC [54]. Furthermore, compared to DC with normoalbuminuria, 8-OHdG content was higher in DKD individuals with macroalbuminuria or chronic renal failure [54]. Makers of oxidative damage were accompanied by some diminished antioxidant capacity. TRX, a small redox protein with ROS scavenging ability, expression was decreased in renal tissue from individuals with DKD compared to NDC. Plasma SOD activity was decreased compared to NDC [63] in one study and unchanged in another [46]. Finally, one study reported decreased expression of sestrin-2, an endogenous antioxidant protein, in glomerular podocytes from individuals with DKD compared to NDC [65].

### 3.4. Mitochondrial Respiration, Uncoupling and Membrane Potential

To determine whether altered mtDNA content affected mitochondrial respiratory capacity and glycolysis, one study assessed mitochondrial oxygen consumption rate, extracellular acidification rate, and bioenergetic health index in freshly isolated PBMCs (Table 5).

Maximal and reserve respiratory capacities were decreased (~40%) in live PBMCs from individuals with DKD compared to DC, while basal respiration, ATP-linked respiration, and basal glycolytic rate were unchanged [56]. Individuals with DKD also had less metabolic flexibility (i.e., the ability to rapidly adapt to an acute stress). When treated with an acute glucose load (20 mM), DKD PBMCs had decreased maximal and reserve mitochondrial respiration compared to normal glucose, but acute glucose treatment did not affect NDC and DC PBMCs. 

Mitochondrial membrane potential and uncoupling were investigated in two studies (Table 5) [17,45]. MicroRNAs miR-15a-5p and miR-30e-5p regulate uncoupling protein 2 (UCP2) gene expression. In individuals with severe DKD, both plasma and urine miR-30e-5p were downregulated compared to individuals with diabetes but no renal disease [45], while in moderate DKD only plasma miR-30e-5p was decreased. miR-15a-5p was unchanged in plasma and urine. PBMCs isolated from individuals with DKD had decreased mitochondrial membrane potential compared to NDC (*p* = 0.024) [17]. 

## 4. Discussion

Mitochondrial dysfunction is implicated in the development and progression of DKD; however, there is a paucity of clinical data available supporting this paradigm when compared with preclinical in vitro and in vivo studies. In the current review, we performed a systematic search of the literature to determine whether individuals with DKD have evidence of mitochondrial dysfunction. Samples from individuals with DKD (including renal biopsy, muscle biopsy, blood, plasma, serum, isolated cells, and urine) provided evidence of mitochondrial dysfunction, as demonstrated by altered mtDNA content, mtDNA damage, disrupted mitochondrial dynamics, oxidative damage, decreased antioxidant capacity, and decreased mitochondrial respiratory capacity. Of the 22 articles included, most studies (96%) reported biological markers that do not directly reflect mitochondrial function, and only one study directly assessed mitochondrial function (i.e., mitochondrial respiratory capacity). Taken together, these findings suggest that systemic mitochondrial dysfunction is present in individuals with DKD.

The link between mtDNA mutations, diabetes and renal pathology is clearly demonstrated in individuals with genetic mitochondrial diseases [66], where diabetes is the most commonly reported endocrinopathy in individuals with mitochondrial disease [67]. We identified that individuals with DKD had systemic mtDNA damage and decreased circulating mtDNA content. Furthermore, stratification by degree of albuminuria demonstrated that decreased mtDNA content and increased oxidative damage were associated with the severity of DKD, establishing a link between mitochondrial dysfunction and DKD progression. Urinary exosome mtDNA content was also decreased with DKD, indicating a reduction in intracellular mtDNA in glomerular and tubular epithelial cells. This leads us to postulate that individuals with DKD have a systemic mtDNA deficiency and acquired mtDNA damage. Renal tubule cells with dysfunctional mitochondria can release mtDNA into the urine, and urinary mtDNA is inversely correlated with eGFR and positively correlated with interstitial fibrosis in established DKD [68]. In agreement, urinary mtDNA/creatinine ratio was increased in individuals with diabetes, especially those with clinically significant proteinuria [48]. Most studies reported decreased eGFR in individuals with DKD, implying that they were in later stages of the disease and so increased urinary mtDNA could not be explained by greater urinary output driven by renal hyperfiltration. We, therefore, postulate two alternatives; (1) that renal cells with dysfunctional mitochondria release extracellular mtDNA directly into the urine, and/or (2) a greater number of dying renal cells are shed into the urinary filtrate in individuals with overt DKD, releasing mtDNA into the urine due to cell lysis due to the osmotic and pH differences in the urine. These events would perpetuate renal mtDNA deficiencies, mitochondrial impairment, and cellular stress. However, we note that in addition to mtDNA content, we must also appreciate the functional state of mitochondria and the factors that contribute to mtDNA content, which are discussed below.

Hyperglycaemia modulates renal mitochondrial damage via phosphorylation and oxidation of mitochondrial proteins, production of ROS, decreased antioxidant capacity, and increased advanced glycation end-products (AGEs) [69,70,71,72], and could explain mtDNA damage and decreased circulating mtDNA content. Congruent with diabetes diagnosis, the majority of studies reported elevated HbA_1c_ in individual with diabetes regardless of kidney disease. Therefore, if poor glycaemic control was the primary regulator of mtDNA content/damage, then we would expect similar changes in mtDNA content/damage in DC and DKD. However, three studies reported decreased mtDNA content in DKD compared to DC. Another possibility is that decreased mtDNA content and mtDNA damage was due to oxidative damage. Indeed, individuals with DKD had increased markers of oxidative damage compared to NDC and DC. TRX expression, sestrin-2, and superoxide dismutase were decreased in DKD compared to NDC, while manganese superoxide dismutase was not significantly altered compared to DC. These findings suggest that renal antioxidant capacity is decreased in diabetes regardless of kidney disease status and likely contributes to mtDNA damage, but it does not explain the functional differences between DC and DKD. In agreement, there has been limited end-organ protection afforded by antioxidant therapies in clinical trials to date [5]. 

Increased mtDNA content is considered an acute adaptive response to oxidative stress (i.e., via increased mitochondrial biogenesis), whereas chronic oxidative stress results in mtDNA damage and decreased content [20,73]. Mitochondrial biogenesis is a major regulator of mtDNA content, capacity to generate ATP, and defence against oxidative damage. Four studies reported decreased mitochondrial biogenesis in individuals with DKD, indicating that decreased biogenesis could contribute to decreased mtDNA content and reduced capacity to respond to oxidative stress. Of note, one study reported increased circulating mtDNA content in individuals with DKD; however, this study did not report any markers of oxidative stress or biogenesis; therefore, it was not possible to conclude whether oxidative stress was associated with increased mtDNA content [52]. Furthermore, given the population characteristics (i.e., age and duration of diabetes) were similar to the other studies, it would seem unlikely that an “acute” response could account for these differences. A more likely explanation was previously proposed, where mitochondrial primers could co-amplify nuclear pseudogenes with high level of homology to mtDNA, resulting in the observed increased mtDNA levels in individuals with DKD [73]. Other methodological variations could also affect mtDNA values, specifically in PBMCs, where leukocyte proportion is considered an influencer of mtDNA content and should be taken into consideration. Furthermore, whether it is more accurate to correct urinary mtDNA content for creatinine or nuclear DNA is unresolved. This underscores the importance of developing robust and reproducible methodologies for measuring mtDNA, especially if it were to be adopted as a clinically applicable biomarker in the future. 

Disruption of mitochondrial dynamics results in the accumulation of damaged mitochondria and perpetuates ATP imbalances [13], where exposure to excess nutrients promotes decreased fusion and increased fission and is linked to mitochondrial uncoupling [18]. In agreement, individuals with DKD had increased renal mitochondrial fission and fragmentation. Hyperglycaemia induced mitochondrial fission and fragmentation mediated increased ROS production, mitophagy and apoptosis [74,75]. Additionally, decreased MFN2 was identified in renal biopsy but not in peripheral blood of individuals with DKD. As part of quality control mechanisms to remove mtDNA genomes with pathological mutations and deletions (i.e., when the mutation is less than ~85%), mitochondria within the same cell have the ability to fuse together to produce daughter mitochondria which contain defective components for removal by mitophagy and to maximise oxidative capacity under pathological stress [9]. The complete absence of mitochondrial fusion in mouse embryo fibroblasts (derived from double knockout of Mfn1 Mfn2) resulted in decreased mtDNA content, a loss of membrane potential, and impaired ATP production [76] which could explain the relationship between decreased mitochondrial fusion and mtDNA content in DKD. Therefore, we postulate that decreased renal mitochondrial fusion may have contributed to the presence/accumulation of damaged mtDNA, decreased circulating/intracellular mtDNA content and oxidative capacity. 

Disturbances in renal oxygenation and ATP production are widely reported in the diabetic kidney, where ATP is elevated in early disease and subsequently declines with disease progression [41,77,78,79]. Recent studies have underscored the relationship between oxygen tension and mitochondrial ATP production and proposed that increased mitochondrial oxygen consumption may contribute to renal hypoxia and fibrosis [40,80]. In the present review, individuals with DKD exhibited decreased mitochondrial respiratory capacity (i.e., maximal respiration, reserve capacity, and bioenergetic health index), as well as a loss of metabolic flexibility in response to metabolic stress (i.e., an acute glucose load). Therefore, decreased mtDNA content, mtDNA damage, oxidative stress and altered mitochondrial dynamics may confer decreased oxidative capacity and highlights functional mitochondrial differences between individuals with a long duration of diabetes (i.e., ~22 years), with and without DKD. Further research is needed to elucidate the temporal changes occurring in the diabetic kidney and whether increased renal mitochondrial oxygen consumption in early disease contributes to renal hypoxia and fibrosis. This should include investigating whether mitochondrial abnormalities are present in early diabetes, ideally prior to clinically evident kidney disease, to elucidate whether mitochondrial dysfunction is a cause or consequence of DKD. 

Dysregulation of uncoupling pathways is evidenced in diabetes [5]. Dysregulation of miR-15a-5p and miR-30e-5p (i.e., microRNAs that target the mitochondrial UCP2 gene) is implicated in DKD, particularly in experimental studies which have demonstrated the link between these miRNAs, podocyte injury and renal fibrosis [45]. In agreement, miR-30e-5p was decreased in plasma from individuals with moderate and severe DKD, and in urine from individuals with severe DKD. The authors concluded, following further bioinformatics analysis, that these miRNAs regulated genes involved in apoptosis, hypoxia, and oxidative stress pathways, further supporting their pathological link to DKD [45]. Additionally, dysregulation of uncoupling pathways was further evidenced in this population by a loss of mitochondrial membrane potential in PBMCs isolated from individuals with DKD [17]. 

Although not a primary outcome of the present review, we note that mitochondrial function was different between NDC and DC groups. If diabetes itself resulted in mitochondrial dysfunction in this population, then we would expect to see a similar change in individuals with diabetes who do not have DKD (i.e., DC group), especially given that no study reported that HbA_1c_ was significantly different between DC and DKD groups. However, differences were reported between DC and DKD across all four categories of mitochondrial dysfunction. These findings reinforce that, while diabetes and glycaemic control undoubtedly influence mitochondrial function, additional pathological mechanisms contribute to and perpetuate mitochondrial dysfunction in DKD. Certainly, seminal studies have demonstrated that strict glycaemic control affords protection against vascular complications, including DKD, but also that glycaemic control cannot eliminate all incidence of disease [81,82,83]. Therefore, individuals who develop DKD may have an underlying susceptibility to mitochondrial dysfunction which may begin as early as diabetes onset and is perpetuated by poor glycaemic control. MtDNA is highly polymorphic, and variations could contribute to susceptibility to DKD, although reviewing mitochondrial polymorphisms associated with DKD was beyond the scope of this study. Furthermore, it is appreciated that glycaemic variability may be more detrimental than sustained hyperglycaemia and confer greater risk for diabetic vascular complications, likely due to dysregulated flux through the respiratory chain, inhibiting adaptation and compensatory mechanisms. Unfortunately, glycaemic variability is rarely reported in human DKD studies and undoubtedly warrants investigation in future studies. 

The present review had some limitations. Whilst the number of studies included in the systematic review was acceptable, the diversity of the mitochondrial markers reported meant that some markers were only represented in a single study, and it was not possible to complete a meta-analysis. Due to the limited number of studies in individuals with T1DM, it was also not possible to ascertain whether mitochondrial dysfunction manifested differently in individuals with T1DM versus T2DM. Furthermore, participant characteristics were rarely reported for renal clinical biopsy samples. While a substantial body of evidence demonstrated the role of altered mitochondrial dynamics in DKD, there is far less evidence pertaining to functional mitochondrial differences (i.e., mitochondrial respiratory capacity and/or ATP production) in clinical samples. Similarly, when considering changes to mitochondrial structure and function it is important to recognise the role of gene expression, transcription, and translation, particularly as it relates to mitochondrial-related genes, and given the rapid advancement of omics this warrants further study. Finally, it should be noted that while the present review focuses on mitochondrial dysfunction in DKD, aberrations to mitochondrial structure and function are widely reported in other forms of CKD and as such it is important to consider the findings of the present systematic review in the wider context of CKD. Adequately establishing the role of mitochondrial dysfunction in CKD has faced similar challenges to those described earlier (i.e., the relationship between mitochondrial dysfunction in CKD can vary depending on the often-ambiguous definition of “mitochondrial dysfunction” itself, in addition to the variability introduced by the numerous models/populations studied) [84]. Yet, despite these challenges, there are hallmark features of mitochondrial dysfunction described in the context of CKD, such as those described in the context of DKD in the present review, including altered mitochondrial structure and remodelling, increased oxidative stress, impaired mitochondrial biogenesis, and marked decreases to ATP production [84]. Further research is needed to adequately characterise and define mitochondrial dysfunction in CKD, as well as the molecular mechanisms underpinning mitochondrial dysfunction, which will undoubtedly offer new insights into diagnostic, management, and treatment options for many forms of CKD, including DKD, in the future.

## 5. Conclusions

Clinical evidence supports the presence of mitochondrial dysfunction in DKD. Neither presence of diabetes nor elevated HbA_1c_ explained altered mitochondrial function and, rather, mitochondrial dysfunction was related to the presence and severity of DKD. Individuals with DKD may have underlying mitochondrial abnormalities that place them at risk of DKD or alternatively; DKD may induce mitochondrial dysfunction. It would be beneficial to design and complete studies in younger individuals with shorter duration of diabetes, ideally prior to clinically evident DKD, to ascertain whether mitochondrial dysfunction is a cause or consequence of DKD. Whether early therapeutic interventions with targeted mitochondrial pharmacotherapies could benefit individuals at risk of DKD should also be explored.

## Figures and Tables

**Table 2:** Summary of mtDNA content.

Study	Sample (Units)	NDC	DC	DKD
Malik [52]	Peripheral blood ^a^ (CytB/B2M)	1379 ± 1572	845 ± 709	3,259 ± 3228 * ^#^
Czajka [56]	Peripheral blood ^b^ (CytB/B2M)	32 (35)	38 (469) *	26 (298) ^###^
Al-Kafaji [47]	Peripheral blood ^c^ (CytB:B2M)	39.75 ± 6.6	35.11 ± 5.4 *	20.85 ± 5.2 ** ^##^
Jiang [17]	Serum ^d^ (ND1: β-actin)	11.03 (6.84)	11.83 (14.37)	9.59 (7.31) ^#^
Cao [48]	Plasma ^e^ (log_10_ copies/µL)	3.49 ± 0.68	3.13 ± 0.66 ^f^ *	
	Urine ^e^ (log_10_ copies/µL/Cr)	vnr	vnr ^g^ **	
Sharma [58]	Urinary exosome ^h^ (copies/ng total DNA)	432 ± 147	n/a	36 ± 18 **

Data are presented as mean ± SD, median (interquartile range). * *p* < 0.05 vs. NDC, ** *p* < 0.01 vs. NDC, ^#^ *p* < 0.05 vs. DC, ^##^ *p* < 0.01 vs. DC, ^###^ *p* < 0.001 vs. DC. ^(a)^ (NDC; *n* = 21), (DC; *n* = 21), (DKD; *n* = 20); ^(b)^ (NDC; *n* = 39), (DC; *n* = 45), (DKD; *n* = 83); ^(c)^ (NDC; *n* = 50), (DC; *n* = 50), (DKD; *n* = 50); ^(d)^ (NDC; *n* = 65), (DC; *n* = 48), (DKD; *n* = 60); ^(e)^ (NDC; *n* = 35), (DM; *n* = 42); ^(f)^ when population was stratified by DKD status (NDC; *n* = 35), (DC; *n* = 23); (DKD; *n* = 19), DKD was significantly decreased (*p* = 0.012) and DC was unchanged (*p* = 0.587) compared to NDC, although values were not reported; ^(g)^ when population was stratified by DKD status (NDC; *n* = 35), (DC; *n* = 23); (DKD; *n* = 19), urine mtDNA/creatinine ratio were significantly increased in both DKD and DC compared to NDC (*p* < 0.001), although values were not reported; ^(h)^ (NDC; *n* = 16), (DKD; *n* = 16). B2M, beta-2 microglobulin; Cr, creatinine; CytB, Cytochrome B; DC, diabetic control without DKD; DKD, diabetic kidney disease; DM, diabetes mellitus; DNA, deoxyribonucleic acid; NDC, non-diabetic control; ND1, NADH-ubiquinone oxidoreductase chain 1 protein; vnr, mtDNA value not reported and only level of significance reported in original research article.

**Table 3:** Summary of mitochondrial dynamics (i.e., fission, fragmentation, fusion, apoptosis, mitophagy, biogenesis) in individuals with DKD compared to NDC and/or DC.

Study	Sample	Marker	vs. NDC	vs. DC
Fission/Fragmentation				
Czajka [56]	Peripheral blood	DRP1 ^a^	↓, ns	↓, ns
Han [59]	Biopsy	Fragmentation ^b^	↑, *p* < 0.05	—
Jiang [17]	Biopsy	Fragmentation (tubules) ^c^	↑, *p* = 0.017	—
		Fragmentation (podocytes) ^c^	⇌, ns	—
		DRP1 ^c^	↑, *p* = 0.025	—
		FIS1 ^c^	↑, *p* = 0.044	—
Ma [62]	Biopsy	Fragmentation ^d^	↑, *p* < 0.001	—
		DRP1 ^d^	↑, nq	—
		AKAP1 ^d^	↑, nq	—
**Fusion**				
Czajka [56]	Peripheral blood	Mitofusin 1 ^a^	⇌, ns	⇌, ns
		Mitofusin 2 ^a^	⇌, ns	⇌, ns
		Optic atrophy 1 ^a^	↓, ns	↓, ns
Ding [64]	Biopsy	Mitofusin 2 ^e^	↓, nq	—
Jiang [17]	Biopsy	Mitofusin 2 ^c^	↓, *p* = 0.014	—
**Apoptosis**				
Horne [60]	Biopsy	KLF6 (glomerular) ^f^	↓, *p* < 0.01^late^	—
		KLF6 (podocyte) ^f^	↓, *p* < 0.01 ^late^	—
		KLF6 (podocyte) ^f^	↓, *p* < 0.01 ^early^	—
Jiang [17]	PBMCs	Apoptotic cells ^g^	↑, *p* = 0.002	↑, *p* = 0.032
	Biopsy	Apoptotic cells ^h^	↑, *p* < 0.0001	
		BAX ^h^	↑, *p* < 0.0001	—
		Cytochrome C ^h^	↑, *p* = 0.030	—
Sharma [58]	Biopsy-1	Cytochrome C ^i^	↓, *p* < 0.05	—
**Mitophagy**				
Czajka [56]	Peripheral blood	PINK ^a^	↓, *p* < 0.05	⇌, ns
		PARK2 ^a^	⇌, ns	↑, *p* < 0.05
**Biogenesis**				
Czajka [56]	Peripheral blood	Transcription factor A ^a^	↓, ns	↑, *p* < 0.05
		PGC1- α ^a^	⇌, ns	⇌, ns
Li [49]	Biopsy	PGC1-α ^j^	↓, *p* < 0.01	↓, *p* < 0.05
		*p*-AMPK ^j^	↓, *p* < 0.01	↓, *p* < 0.01
Li [61]	Biopsy	PPARGC1A ^k^	↓, *p* = 0.0009	—
		PGC1-α ^k^	↓, nq	—
Li [50]	Biopsy	PGC1-α ^l^	↑, *p <* 0.05	—
Sharma [58]	Biopsy-2	PGC1-α ^m^	↓, *p* < 0.05	—
Zhou [55]	Biopsy	Progranulin ^n^	↓, *p* < 0.05	↓, *p* < 0.05

^(a)^ (NDC; *n* = 39), (DC; *n* = 45), (DKD; *n* = 83); ^(b)^ (NDC; *n* = 15), (DKD; *n* = 15); ^(c)^ (NDC; *n* = 3), (DKD; *n* = 3); ^(d)^ (NDC; *n* = 6), (DKD; *n* = 31); ^(e)^ (NDC; *n* = 6), (DKD; *n* = 6); ^(f)^ (NDC; *n* = 12), (DKD^early^; *n* = 7), (DKD^late^; *n* = 17); ^(g)^ (NDC; *n* = 9), (DC; *n* = 19), (DKD; *n* = 14); ^(h)^ (NDC; *n* = 15), (DKD; *n* = 14); ^(i)^ (NDC; *n* = 5), (DKD; *n* = 5); ^(j)^ (NDC; *n* = 5), (DC; *n* = 5), (DKD; *n* = 5); ^(k)^ (NDC; *n* = 14), (DKD; *n* = 7); ^(l)^ (NDC; *n* = 10), (DKD; *n* = 33); ^(m)^ (NDC; *n* = 8), (DKD; *n* = 14); ^(n)^ (NDC; *n* = 8), (DC; *n* = 7), (DKD; *n* = 8). AKAP1, A-kinase anchor protein 1; BAX, Bxl-2-associated X protein; DC, diabetic control without DKD; DKD, diabetic kidney disease; DRP1, Dynamin-related protein 1; FIS1, Mitochondrial fission protein 1; KLF6, Kruppel-like factor 6; NDC, non-diabetic control; ⇌, no change; ns, not significant; p-AMPK, phosphorylated AMP-activated protein kinase; PBMCs, peripheral blood mononuclear cells; PGC1-α, peroxisome proliferator-activated receptor gamma coactivator 1-alpha; PPARGC1A, peroxisome proliferator-activated receptor gamma coactivator 1-alpha gene.

**Table 4:** Summary of oxidative damage and antioxidant profile in individuals with DKD compared to NDC and/or DC.

Study	Sample	Marker	vs. NDC	vs. DC
**Oxidative Damage**				
Han [59]	Biopsy	Superoxide ^a^	↑, *p* < 0.05	—
Jiang [17]	PBMCs	H_2_DCFDA ^b^	↑, *p* = 0.038	↑, *p* = 0.024
	Biopsy	Nitrotyrosine ^c^	↑, *p* = 0.010	—
		Nox4 ^c^	↑, *p* = 0.002	—
Li [50]	Biopsy	AOPP ^d^	↑, *p* < 0.05	—
		8-OHdG ^d^	↑, *p* < 0.05	—
Li [51]	Biopsy	AOPP ^e^	↑, *p* < 0.001	—
Mohammedi [46]	Plasma	AOPP ^f^	—	↑, *p* = 0.03 ^k^
Qi [57]	Urine	8-oxo-G/Cr ^g^	—	↑, *p* < 0.01 ^progressive^
		8-oxo-G/Cr ^g^	↑, *p* < 0.05 ^non-progressive vs. progressive^	
	Biopsy	8-oxo-G ^h^	↑, nq ^mod-severe^	—
		8-oxo-G ^h^	↑, nq ^advanced^	
Qin [63]	Plasma	Malondialdehyde ^i^	↑, *p* < 0.05	—
Suzuki [54]	Muscle biopsy	8-OHdG ^j^	↑, *p* < 0.02 ^micro^	⇌, ns ^micro^
		8-OHdG ^j^	↑, *p* < 0.0001 ^macro^	↑, *p* < 0.02 ^macro^
		8-OHdG ^j^	↑, *p* < 0.0001 ^crf^	↑, *p* < 0.0002 ^crf^
**Antioxidant Capacity**				
Ding [65]	Biopsy	Sestrin-2 ^l^	↓, nq	—
Han [59]	Biopsy	TRX expression ^a^	↓, *p* < 0.05	—
Qin [63]	Plasma	SOD activity ^i^	↓, *p* < 0.05	—
Mohammedi [46]	Plasma	SOD activity ^f^	—	⇌, ns ^m^

^(a)^ (NDC; *n* = 15), (DKD; *n* = 15); ^(b)^ (NDC; *n* = 3), (DC; *n* = 7), (DKD; *n* = 4); ^(c)^ (NDC; *n* = 15), (DKD; *n* = 14); ^(d)^ (NDC; *n* = 10), (DKD; *n* = 33); ^(e)^ (NDC; *n* = 10), (DKD; *n* = 34); ^(f)^ (DC; *n* = 131), (DKD^incipient^; *n* = 83), (DKD^advanced^; *n* = 167); ^(g)^ (DC; *n* = 22), (DKD^non-progressive^; *n* = 14), DKD^intermediate^; *n* = 23), (DKD^progressive^; *n* = 12); ^(h)^ glomerular endothelial cells (DC; *n* = 6), (T2DM + mild-moderate DKD; *n* = 7), (T1DM + mild-moderate DKD; *n* = 1), (T2DM + moderate-severe DKD; *n* = 5), (T2DM + advanced DKD; *n* = 1); ^(i)^ (NDC; *n* = 15), (DKD; *n* = 20); ^(j)^ (NDC; *n* = 7), (DC; *n* = 6), (DKD^micro^; *n* = 4), (DKD^macro^; *n* = 7), (DKD^crf^; *n* = 5); ^(k)^ DC versus DKD^advanced^, DKD^incipient^ versus DKD^advanced^, by ANCOVA adjusted for sex, age, HbA_1c_ and use of ACE inhibitors; ^(l)^ (NDC; *n* = 6), (DKD; *n* = 6); ^(m)^ ANCOVA adjusted for sex, age and use of ACE inhibitors. AOPP, advanced oxidation protein products; Cr, creatinine; crf, chronic renal failure; DC, diabetic control without DKD; DKD, diabetic kidney disease; H_2_DCFDA, 2′,7′-dichlorodihydrofluorescein diacetate; macro, macroalbuminuria; micro, microalbuminuria; ⇌, no change; NDC, non-diabetic control; nq, not quantified; not significant; PBMCs, peripheral blood mononuclear cells; SOD, superoxide dismutase; TRX, thioredoxin; 8-OHdG, 8-hydroxydeoxyguanosine; 8-oxoG, 8-oxo-deoxyguanosine.

**Table 5:** Summary of mitochondrial respiratory capacity and membrane potential in individuals with DKD compared to NDC and/or DC.

Study	Sample	Marker	vs. NDC	vs. DC
Respiratory capacity				
Czajka [56]	PBMCs	Basal respiratory capacity ^a^	⇌, ns	⇌, ns
		ATP-linked respiratory capacity ^a^	⇌, ns	⇌, ns
		Maximal respiratory capacity ^a^	⇌, ns	↓, *p* < 0.05
		Reserve respiratory capacity ^a^	⇌, ns	↓, *p* < 0.05
		Basal glycolytic rate ^a^	⇌, ns	⇌, ns
		Bioenergetic health index ^b^	⇌, ns	↓, *p* < 0.05
		Basal respiratory capacity ^c^	⇌, ns ^NDC±acute glucose load^	
		ATP-linked respiratory capacity ^c^	⇌, ns ^NDC±acute glucose load^	
		Maximal respiratory capacity ^c^	⇌, ns ^NDC±acute glucose load^	
		Reserve respiratory capacity ^c^	⇌, ns ^NDC±acute glucose load^	
		Basal respiratory capacity ^c^	⇌, ns ^DC±acute glucose load^	
		ATP-linked respiratory capacity ^c^	⇌, ns ^DC±acute glucose load^	
		Maximal respiratory capacity ^c^	⇌, ns ^DC±acute glucose load^	
		Reserve respiratory capacity ^c^	⇌, ns ^DC±acute glucose load^	
		Basal respiratory capacity ^c^	⇌, ns ^DKD±acute glucose load^	
		ATP-linked respiratory capacity ^c^	⇌, ns ^DKD±acute glucose load^	
		Maximal respiratory capacity ^c^	↓, *p* < 0.05 ^DKD±acute glucose load^	
		Reserve respiratory capacity ^c^	↓, *p* < 0.05 ^DKD±acute glucose load^	
**Uncoupling/Membrane potential**				
Dieter [45]	Plasma	miR-15a-5p ^d^	—	⇌, ns
		miR-30e-5p ^d^	—	↓, *p* < 0.05 ^moderate^
		miR-30e-5p ^d^	—	↓, *p* < 0.05 ^severe^
	Urine	miR-15a-5p ^d^	—	⇌, ns
		miR-30e-5p ^d^	—	↓, *p* < 0.05 ^severe^
Jiang [17]	PBMCs	Membrane potential (JC-1) ^e^	↓, *p* = 0.024	↓, ns

^(a)^ (NDC; *n* = 10), (DC; *n* = 14), (DKD; *n* = 16); ^(b)^ (NDC; *n* = 8), (DC; *n* = 5), (DKD; *n* = 12); ^(c)^ (NDC + acute glucose load; *n* = 4), (DC + acute glucose load; *n* = 4), (DKD + acute glucose load; *n* = 8) ^(d)^ (DC; *n* = 17), (DKD^moderate^; *n* = 12), (DKD^severe^; *n* = 11), ^(e)^ (NDC; *n* = 5), (DC; *n* = 4), (DKD; *n* = 3). ATP, adenosine triphosphate; DC, diabetic control without DKD; DKD, diabetic kidney disease; JC-1, tetraethylbenzimidazolylcarbocyanine iodide; ⇌, no change; NDC, non-diabetic control; ns, not significant; PBMCs, peripheral blood mononuclear cells.

## Data Availability

Not applicable.

## Appendix A

**Table A1:** Modified BIOCROSS quality rating tool.

Domain	IC	Recommendation	Included/Excluded	Changes/Justification
Study Rational				
Hypothesis/Objective	1	1.1 Was the biomarker under study described?	Included	Was the outcome measure of mitochondrial dysfunction described?
		1.2 Was the rational for the study (research questions) clearly stated?	Included	
		1.3 Were the study objectives/hypothesis clearly stated?	Included	
**Design/Methods**				
Study population selection	2	2.1 Were the characteristics of the study population presented?	Included	
		2.2 Were the disease stages or comorbidities of the included participants described?	Included	Were diabetes and DKD status of the included participants described?
		2.3 Were the inclusion and exclusion criteria for study participation defined?	Included	
Study population representativeness	3	3.1 Was the sampling frame reported (study population source)?	Included	Was the sampling frame reported for samples used to assess mitochondrial dysfunction?
		3.2 Was the participation rate reported (i.e., eligible persons at least 50%)?	Excluded	Not applicable as the only outcome of interest was group differences between NDC, DC, and DKD.
		3.3 Was sample size justification or power description provided?	Excluded	Not applicable for analysis.
**Data analysis**				
Study population characteristics	4	4.1 Were the study population characteristics (i.e., demographic, clinical, and social) presented?	Excluded	Considered to repeat 2.1.
		4.2 Were the exposures and potential confounders described?	Included	
		4.3 Were any missing values and strategies to deal with missing data reported?	Excluded	Not applicable for analysis.
Statistical analysis	5	5.1 Did the authors clearly report statistical methods used to calculate estimates (e.g., Spearman/Pearson/Linear Regression, etc.)?	Included	Were statistical methods used to identify group differences clearly reported?
		5.2 Were key potential confounding variables measured and adjusted statistically in reported analysis?	Excluded	Not applicable for analysis.
		5.3 Was the raw effect size estimate (correlation coefficient, beta coefficient) or measure of study precision provided (e.g., confidence intervals, precise (1) *p*-value)?	Included	Was the *p*-value reported to distinguish group differences?
**Data interpretation**				
Interpretation and evaluation of results	6	6.1 Was the data discussed in the context of study objectives/hypothesis?	Included	
		6.2 Was the interpretation of the results considering findings from similar studies?	Included	
		6.3 Was the biological context described?	Included	
Study limitations	7	7.1 Was the cross-sectional nature of the analysis discussed	Excluded	Not applicable as the only outcome of interest was group differences between NDC, DC, and DKD.
		7.2 Did the authors acknowledge restricted interpretation due to measurements at one point in time and no statement about causality possible using cross-sectional studies?	Excluded	Not applicable as the only outcome of interest was group differences between NDC, DC, and DKD, not possible causality.
		7.3 Did the authors acknowledge need for consistency with other research?	Included	
**Biomarker measurement**				
Specimen characteristics and assay methods	8	8.1 Were the measurement methods described? (assay methods, preservation and storage, detailed protocol, including specific reagents or kits used)	Included	Were the method(s) used to assess mitochondrial dysfunction adequately described?
		8.2 Were the reproducibility assessments performed for evaluating biomarker stability?	Excluded	Not applicable as the only outcome of interest was group differences between NDC, DC, and DKD.
		8.3 Were the quantitation methods well described?	Excluded	Not applicable for analysis.
Laboratory measurement	9	9.1 Was the laboratory/place of measurement mentioned?	Included	
		9.2 Were any quality control procedures and results reported (e.g., reported coefficient of variation?	Excluded	Not applicable as the only outcome of interest was group differences between NDC, DC, and DKD.
		9.3 Were the analysis blinded for laboratory staff?	Included	
Biomarker data modelling	10	10.1 Was the distribution of biomarker data reported (if non-normal how was it standardised)?	Excluded	Not applicable as the only outcome of interest was group differences between NDC, DC, and DKD.
		10.2 Did the authors report on methods or outlier detection and handling?	Included	
		10.3 Were any possible errors resulting from measurement inaccuracies discussed?	Included	

DC, diabetic control without DKD; DKD, diabetic kidney disease; IC, Issues to consider; NDC non-diabetic control.
